# Lysine-specific demethylase LSD1 regulates autophagy in neuroblastoma through SESN2-dependent pathway

**DOI:** 10.1038/onc.2017.267

**Published:** 2017-08-07

**Authors:** S Ambrosio, C D Saccà, S Amente, S Paladino, L Lania, B Majello

**Affiliations:** 1 Division of Genetics, Department of Biology, University of Naples, ‘Federico II’, Naples, Italy; 2 Department of Molecular Medicine and Medical Biotechnologies, University of Naples, ‘Federico II’, Naples, Italy; 3 Ceinge Biotecnologie Avanzate s.c.a.r.l., Naples, Italy

## Abstract

Autophagy is a physiological process, important for recycling of macromolecules and maintenance of cellular homeostasis. Defective autophagy is associated with tumorigenesis and has a causative role in chemotherapy resistance in leukemia and in solid cancers. Here, we report that autophagy is regulated by the lysine-specific demethylase LSD1/KDM1A, an epigenetic marker whose overexpression is a feature of malignant neoplasia with an instrumental role in cancer development. In the present study, we determine that two different LSD1 inhibitors (TCP and SP2509) as well as selective ablation of LSD1 expression promote autophagy in neuroblastoma cells. At a mechanistic level, we show that LSD1 binds to the promoter region of Sestrin2 (SESN2), a critical regulator of mTORC1 activity. Pharmacological inhibition of LSD1 triggers SESN2 expression that hampers mTORC1 activity, leading to enhanced autophagy. SESN2 overexpression suffices to promote autophagy in neuroblastoma cells, while loss of SESN2 expression reduces autophagy induced by LSD1 inhibition. Our findings elucidate a mechanism whereby LSD1 controls autophagy in neuroblastoma cells through SESN2 transcription regulation, and we suggest that pharmacological targeting of LSD1 may have effective therapeutic relevance in the control of autophagy in neuroblastoma.

## Introduction

Cancerous cells must deal with effective mechanisms of cell death, thereby reducing activation of defense pathways in response to oncogenic insults.^1, 2^ The induction of apoptosis is the major cause route of cell death yet multiple cellular processes, including autophagy, antagonize it.

Autophagy is a conserved intracellular process in which cytoplasmic components are degraded within lysosomes having a central role in cell metabolism and homeostasis. There are different types of autophagy: micro-autophagy, selective autophagy, macro-autophagy and chaperone-mediated autophagy.^3^ Macro-autophagy is the main autophagic pathway and consists in the formation of double-membrane autophagosomes that sequester cellular components and then fuse with lysosomes for degradation and recycling of macromolecules and organelles. Autophagy normally operates at low, basal levels in cells but can be strongly induced by cellular stress. Defective autophagy is associated with human pathologies such as bacterial and viral infections, neurodegenerative diseases and cancer.^4, 5, 6^

Autophagy has dual roles in cancer; it can function as a tumor suppressor, by preventing the accumulation of damaged proteins and organelles, or a survival pathway, by impairing apoptosis and promoting the growth of tumor growth.^7, 8, 9^ Recent studies showed that autophagy has a causative role in chemotherapy resistance in leukemia^10^ and in solid cancers.^7, 10^ Nonetheless, the molecular mechanisms underlying the autophagy on tumorigenesis must be further investigated.

Mammalian target of rapamycin complex 1 (mTORC1) is the major regulator of autophagy. In the presence of nutrients, mTORC1 is activated, resulting in inhibition of the Ulk1 complex and repression of autophagy.^11^ Following nutrient deprivation, mTORC1 is inhibited, and Ulk1 complexes can lead autophagosome formation. Given its pivotal role in autophagy regulation, mTORC1 is the main target for drug development to modulate the autophagic pathway.^12, 13^

Recently, several reports demonstrate that autophagy is regulated by epigenetic alterations, as histone methylation and acetylation.^14, 15, 16^ The mechanisms through which cancer-associated epigenetic alterations modulate autophagy have not yet been elucidated. An epigenetic enzyme that has been target of drug discovery is the lysine-specific demethylase 1, LSD1. LSD1 (also known as KDM1A and AOF2) is an amine oxidase that catalyzes lysine demethylation in a flavin adenine dinucleotide-dependent oxidative reaction^17^ and removes mono- and dimethyl groups from lysine K4 and, in specific circumstances, K9 on histone H3.^17, 18, 19^ More recently, it has been shown that the neuron-specific isoform LSD1n has a new substrate specificity, targeting histone H4 Lys 20.^20^ Finally, LSD1 can also target non-histone proteins such as p53, E2F1 and DNMT.^21, 22, 23^ LSD1 has been demonstrated to have important roles in many important aspects of cell biology, such as cell proliferation, cell mobility and differentiation.^24, 25, 26^ Most importantly, LSD1 is overexpressed in a variety of tumors and its high expression correlate with more aggressive cancers with poor prognosis. There is a large body of evidence that LSD1 is involved in maintaining the undifferentiated, malignant phenotype of neuroblastoma (NB) cells and that its overexpression correlates with aggressive disease, poor differentiation and infaust outcome.^24, 27^

In the present study, we define a novel role of the epigenetic regulator LSD1 in the modulation of autophagy. We found that selective ablation of LSD1, or pharmacological inactivation of its catalytic function, inhibits mTORC1 activity enabling enhanced autophagy. Mechanistically, we found that LSD1 binds to the promoter region of Sestrin2 (SESN2) and represses its expression. LSD1 inhibition triggers SESN2 expression that hampers mTORC1 activity leading to Transcription Factor EB (TFEB) nuclear translocation driving the expression of the Coordinated Lysosomal Expression and Regulation regulatory pathway. Taken together, our findings indicate that LSD1 regulates autophagy in NB cells via transcriptional regulation of SESN2 that serves as a key positive regulator of the mTORC1-dependent autophagy pathway.

## Results

### LSD1 inhibition represses the mTORC1 pathway

**Figure 1:**
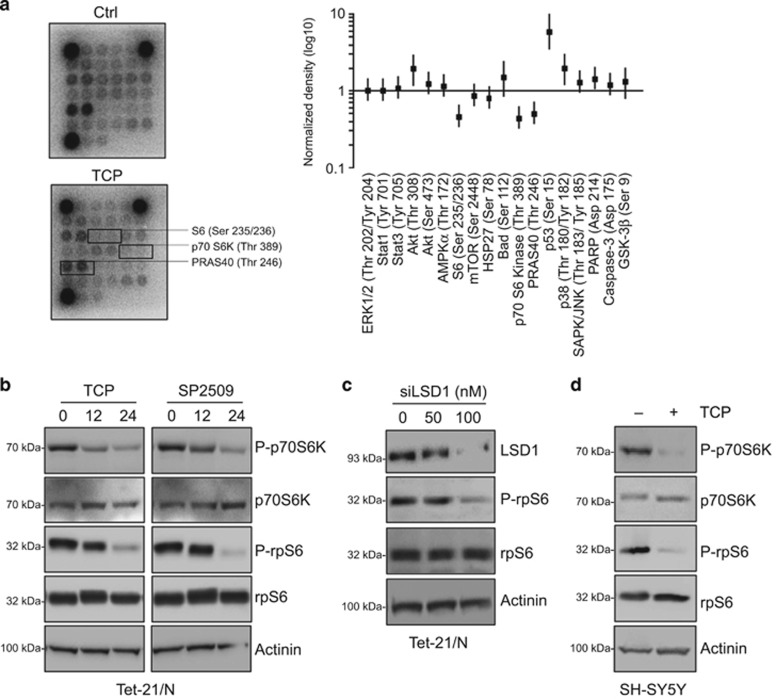
LSD1 inhibition represses mTORC1 activity. (**a**) Protein extract from Tet-21/N treated with 1 mM TCP or vehicle (Ctrl) for 24 h were analyzed for the signaling activation using the PathScan antibody arrays. Graph shows the pixel density ratio of signaling molecule dots. Values represent the means of two independent experiments (± s.d.). (**b**) Western blotting of protein extracts from Tet-21/N cells treated for 0, 12 or 24 h with TCP or SP2509 (1 μM). (**c**) Tet-21/N cells were transfected with siRNA against LSD1 at different concentrations, or scramble (0). Cells were collected 48h after transfection. (**d**) Western blotting of protein extracts from SH-SY5Y cells treated for 24 h with TCP using the indicated antibodies. Actinin was probed as loading control.

LSD1 is highly expressed in undifferentiated NBs and its expression correlates with adverse outcome; however, the molecular mechanism underlying LSD1 effects is largely unknown. We initially undertook an unbiased approach to uncover how cells respond to the loss of LSD1 function looking at signaling alterations caused by treatment with tranylcypromine (TCP), a potent inhibitor of LSD1 in Tet-21/N NB cells. PathScan array was used to determine pathways involved in TCP response. In this assay mTORC1 pathway was the most responsive, evidenced by ribosomal protein S6 (Ser235/236), p70S6 Kinase (Thr389) and PRAS40 (Thr246) phosphorylation reduction (Figure 1a). To verify PathScan array results, we performed western blot analysis of mTORC1 downstream substrates, p70S6K and rpS6. Tet-21/N cells were also treated with SP2509, a reversible inhibitor of LSD1 that, differently from TCP, does not target the catalytic activity of the enzyme, but attenuates the binding of LSD1 to CoREST.^25, 28^ In addition, to address the specific role of LSD1 in mTORC1 activity, we inhibited LSD1 by short interfering RNA (siRNA)-targeted knockdown and measured expression of mTORC1 downstream targets. Protein extracts were prepared at the indicated times and probed with antibodies recognizing phosphorylated and total protein forms of mTORC1 substrates. In agreement with the array data, phosphorylation levels of p70S6K, and consequently of its target rpS6, were downregulated by either TCP or SP2509 treatment as well as in LSD1-KD cells (Figures 1b and c). In addition, similar results were observed in SH-SY5Y NB cells (Figure 1d). Collectively, these findings demonstrated that LSD1 inhibition downregulates mTORC1 signaling.

mTORC1 is known as a critical regulator of autophagy. In response to nutrient deprivation, mTORC1 is inactivated and it dissociates from the Ulk complex, inducing autophagy activation. In addition, mTORC1 has been shown to control autophagy through the functional regulation of the TFEB, a master regulator of lysosomal and autophagic functions. Active mTORC1 phosphorylates and sequestrates TFEB to the cytoplasm; on the contrary, mTORC1 inactivation leads to de-phosphorylation of TFEB, which translocates into the nucleus and drives the expression of lysosomal and autophagy genes that are part of the Coordinated Lysosomal Expression and Regulation regulatory network.^12, 29^

**Figure 2:**
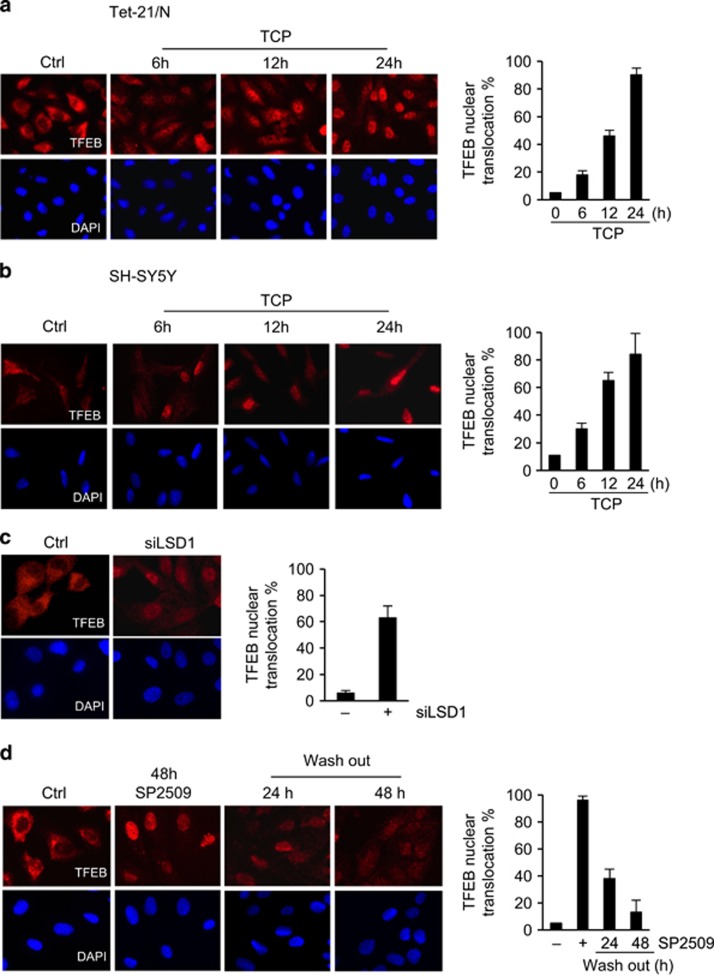
LSD1 inhibition leads to TFEB nuclear sublocalization. Tet-21/N (**a**) and SH-SY5Y (**b**) cells were treated with TCP or vehicle for the indicated times fixed and processed for anti-TFEB immunofluorescence and DAPI staining. (**c**) Tet-21/N cells were transfected with scramble (Ctrl) or siRNA against LSD1 (100 nM), fixed and stained with anti-TFEB and DAPI. (**d**) Tet-21/N cells were treated for 48 h with SP2509 or vehicle and then released into fresh medium for the indicated times before immunostaining with TFEB antibody and DAPI. Graphs show the ratio of TFEB nuclear/cytosolic signal intensity values average, normalized on negative and positive control samples (mean±s.d., *n*=200 cells). DAPI, 4',6-diamino-2-phenylindole.

We sought to investigate the impact of LSD1 inhibition on TFEB subcellular localization. Tet-21/N and SH-SY5Y cells were treated with TCP for the indicated times and analyzed by immunofluorescence using a TFEB antibody. In untreated cells TFEB is localized mainly in the cytoplasm. Consistently with mTORC1 repression, pharmacological inhibition of LSD1 leads to a significant increase of TFEB nuclear levels along with a decreased cytosolic localization (Figures 2a and b). This finding was further confirmed using specific siRNA against LSD1 (Figure 2c).

Because SP2509 is a reversible inhibitor of LSD1, we used this drug to test whether TFEB nuclear shuttling was directly dependent upon LSD1 inhibition. We monitored whether nuclear localization of TFEB was reversed in time after SP2509 wash out. Tet-21/N cells were treated with SP2509 for 48 h and then cells were washed and cultivated in fresh medium for additional 24 and 48 h after drug removal. The immunofluorescence results in Figure 2d show that SP2509 removal decreased the TFEB nuclear/cytoplasmic ratio. Concomitantly, we observed a significant reduction in TFEB levels, suggesting that addition of fresh medium results in degradation of nuclear TFEB.

Similar results have been described using the mTOR inhibitor Torin 1 in MCF7 cells.^30^ These findings demonstrated that TFEB nuclear localization is directly dependent upon LSD1 inhibition.

### LSD1 depletion induces autophagy

**Figure 3:**
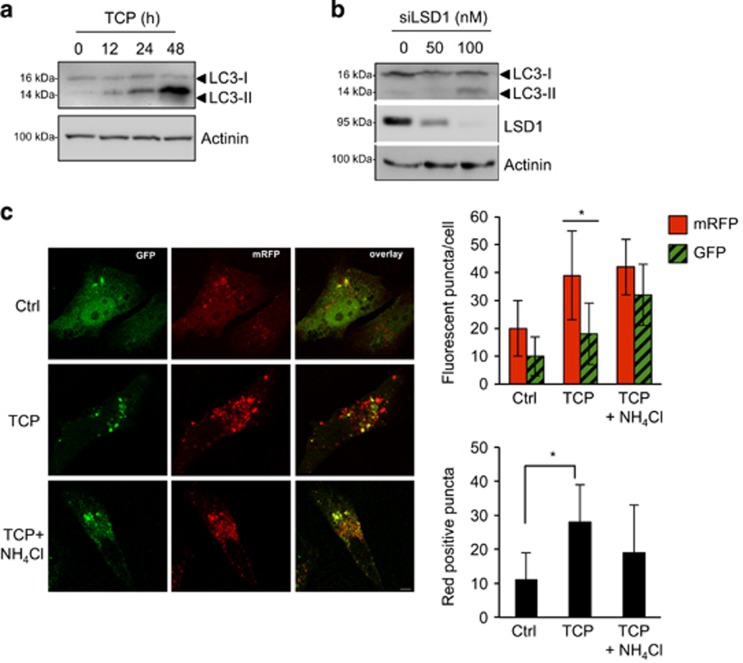
LSD1 inhibition induces autophagy. Tet-21/N cells were treated with TCP for the indicated times (**a**), or siLSD1 at different concentrations as indicated (**b**), and protein extract was prepared and probed using anti-LC3 antibody. LSD1 protein level in cells transfected with siLSD1 or control (0) was shown. Actinin was probed as loading control. (**c**) Tet-21/N was transiently transfected with GFP-mRFP-LC3 tandem construct and treated with TCP alone or in combination with 20 mM NH_4_Cl for 24 h. After treatment, cells were fixed and analyzed by confocal microscopy. Bar, 10µm. Histograms show the number of GFP or mRFP puncta (upper graph) or the number of only-red positive (GFP-negative) puncta (lower graph). The values are expressed as means±s.d. (*n*>100 cells). *P<0.00001.

Autophagy begins with the sequestration of cytosolic proteins in a double-membrane structure called autophagosome. Following fusion with a lysosome, it becomes an autophagolysosome, then lysosomal hydrolases degrade the content of the phagosome that is released in the cytosol for the recycling of macromolecules. During autophagosome formation, the cytosolic form of the microtubule-associated protein 1A/1B-light chain 3 (LC3-I) is conjugated to phosphatidylethanolamine to form lipidated-LC3 (LC3-II), which becomes associated with autophagosomal membranes. Tet-21/N cells were treated with TCP (Figure 3a) or siLSD1-transfected (Figure 3b) and then processed for western blot analysis to monitor the conversion from LC3-I to lower-migrating form LC3-II, as a well-established marker of autophagosome formation.^31^ TCP treatment induced LC3-II form accumulation over time compared with control cells (Figure 3a). Moreover, LSD1-KD also increases the LC3-II form level in a dose-dependent manner (Figure 3b). Similar findings were observed in SH-SY5Y cells (Supplementary Figure 1). Because LC3-II increment might also be interpreted as autophagosome accumulation due to the block of autophagosome–lysosome fusion, we evaluated the autophagic flux following LSD1 inhibition using a green fluorescent protein (GFP)-mRFP-tandem-tagged LC3. Indeed, by taking advantage of the properties of these two fluorescent proteins (GFP signal is quenched inside the acidic lysosomal lumen, but not mRFP) we can discriminate between autophagic compartments before and after fusion with lysosomes.^31, 32^

Tet-21N cells were transiently transfected with the GFP-mRFP-LC3 tandem construct and treated with TCP alone or in combination with NH_4_Cl to block autophagolysosomal degradation by preventing its acidification. In untreated cells, GFP and mRFP signals appear mainly diffused in the cytosol and with few puncta (Figure 3c). Conversely, the number of green and red puncta was higher following TCP treatment, indicating that LSD1 inhibition enhanced the autophagic flux (Figure 3c, upper graph). Moreover, a significant increase in autolysosomes (mRFP-positive, but GFP-negative puncta) was observed upon TCP treatment (Figure 3c, lower graph), indicating that the autophagosome maturation is occurring. Consistently, upon combined treatment of TCP and NH_4_Cl green dots were augmented as expected by the fact that NH_4_Cl, increasing the intralysosomal pH, prevents GFP quenching (Figure 3c).

Taken together, these results clearly indicate that pharmacological inhibition of LSD1 activity triggers a functional autophagic flux induction, as demonstrated by the nuclear localization of autophagy master regulator TFEB and the mature autophagolysosome formation.

### LSD1 inhibition promotes autophagy by increasing SESN2 expression

To understand the mechanisms by which LSD1 inhibition induces autophagy, we analyzed data from our recent published RNA sequencing (RNA-seq) from Tet-21/N cells treated with TCP or siLSD1.^25^ Among the common upregulated genes we identify *SESN2* as an LSD1-repressed target gene. SESN2 is a member of an evolutionarily conserved stress-inducible Sestrin gene family, and it has been shown that SESN2 directly inhibits mTORC1 activity via GATOR2 with a consequent inhibition of mTORC1 recruitment to the lysosomal membrane.^33, 34^ Through these functions, SESN2 serves as a key positive regulator of the autophagic pathway.

**Figure 4:**
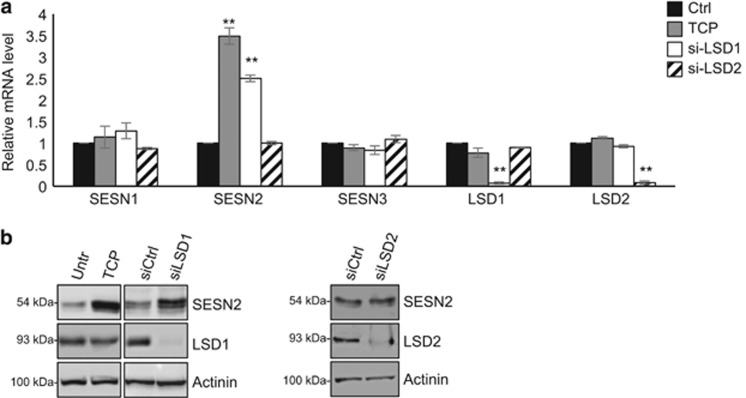
LSD1 represses SESN2 expression. SESN1–3 expression was analyzed by qRT–PCR (**a**) using samples prepared from Tet-21/N cells treated with TCP or siRNA against LSD1 or LSD2 (100 nM). The efficiency of siRNAs to knock down LSD1 and LSD2 expression was shown. (**b**) SESN2 expression was assayed by western blotting, using protein extracts from Tet-21/N cells treated with vehicle, TCP, siRNA-LSD1, siRNA-LSD2 or siRNA-control as indicated. LSD1 and LSD2 protein levels in Tet-21/N cells transfected with specific siRNA or control was analyzed by western blot. Actinin was probed as loading control. **P<0.01.

Although our RNA-seq showed that the relative expression of the other two members of Sestrins, that is, SESN1 and SEN3, were unaffected by treatment with TCP or by siLSD1, we validated RNA-seq data by investigating the consequences of LSD1 inhibition on the relative expression levels of all three members of Sestrins family. Accordingly, with our RNA-seq data, we found by qRT–PCR (quantitative reverse transcriptase polymerase chain reaction) analyses that LSD1 inhibition or silencing enhances SESN2 expression (Figures 4a and b) and only marginally affects SESN1 and SESN3 (Figure 4a), suggesting that SESN2 is predominantly regulated by LSD1 in Tet-21/N cells. However, we cannot exclude that LSD1 might regulate SESN1 and 3 in different cellular backgrounds.

In addition to LSD1, the LSD2 (KDM1B) mammalian paralog also demethylates mono- and di-methylated H3K4 in an FAD-dependent manner. Thus, TCP treatment may also affect LSD2 function. To determine the relative contribution of LSD2 in Sestrins expression, Tet-21/N cells were treated with siRNA against LSD2 and Sestrins mRNA expression was assayed by qRT–PCR. As shown in Figures 4a and b, we found that siLSD2 treatment did not affect SESN2 expression. We conclude that LSD1 specifically affects SESN2 expression.

**Figure 5:**
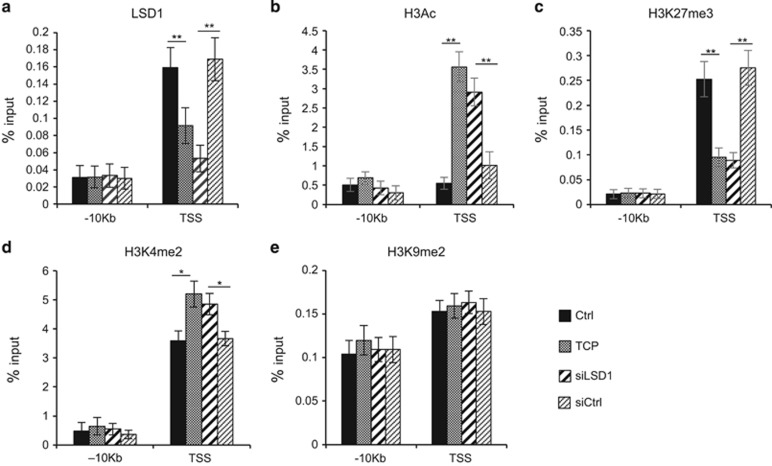
LSD1 binds *SESN2* gene promoter. (**a**) LSD1 binding to SESN2 chromatin. qPCR was performed with primers for SESN2 TSS and −10 kb. (**b**–**e**) Histone modifications at SESN2 chromatin; ChIPs were carried out using the indicated antibodies and analyzed with primers encompassing the TSS region and −10 kb from TSS. Values from three independent ChIP assays are presented with s.d.'s, *n*=3. Changes in % input are shown normalized over IgG controls (*P<0.05; **P<0.01; Student's *t-*test).

Next, we sought to determine whether LSD1 is directly involved in control of *SESN2* gene expression. Public available chromatin immunoprecipitation (ChIP)-seq LSD1 data from SH-SY5Y cells as well as from mouse ES cells indicate a putative LSD1 binding to the promoter region of SESN2 (Supplementary Figure 2). We then carried out ChIP assays to determine binding of LSD1 to SESN2 chromatin. LSD1 binding was analyzed in Tet-21/N cells treated with TCP or silenced for LSD1 expression. Chromatin isolated from Tet-21/N cells was immunoprecipitated with an anti-LSD1 antibody and qPCR analysis performed using primers corresponding to the 5’ regulatory regions surrounding the transcription start site (TSS) of the *SESN2* gene. As shown in Figure 5a, LSD1 is recruited selectively at TSS of the *SESN2* gene but not at distal sites (−10 kb), indicating that LSD1 binds to the SESN2 promoter. LSD1 binding was reduced in TCP-treated and siLSD1 samples, suggesting that binding of LSD1 required catalytic activity; moreover, TCP treatment did not alter the relative LSD1 expression levels (Figures 4a and b).

To better understand how LSD1 can affect chromatin organization at the SESN2 promoter, we analyzed four different histone modifications, H3 pan-acetyl (H3Ac), H3K27Me3, H3K4Me2 and H3K9Me2, around the TSS promoter region. Figure 5b shows that both LSD1 silencing (siLSD1) and inhibition by TCP determine a significant increase in H3 acetylation. As a marker of transcriptional repression, we analyzed lysine 27 tri-methylation of Histone H3. Data presented in Figure 5c demonstrate that both LSD1 silencing or its inhibition (TCP) determine an almost 2.5-fold decrease of the marker. ChIP assays were also performed on di-methylated Lysine 4 of histone H3 (Figure 5d), and we found a significant increase in H3K4me2 at SESN2 TSS following LSD1 inhibition. Conversely, both inhibition and repression of LSD1 do not affect H3K9Me2 signature at TSS level of SESN2 (Figure 5e). These findings highlight the critical role of LSD1 in the transcriptional regulation of the *SESN2* gene through direct binding to SESN2 promoter.

**Figure 6:**
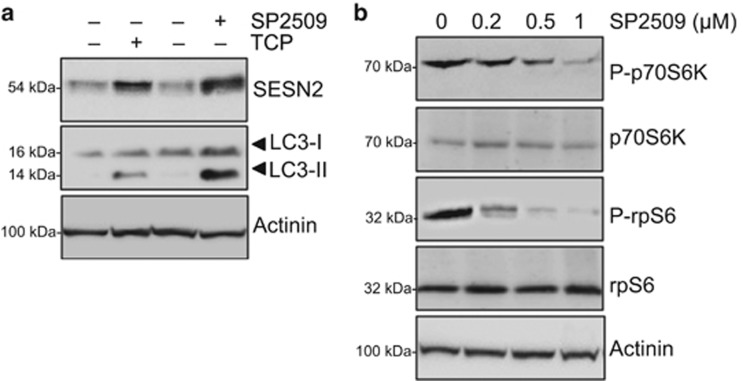
LSD1 depletion-mediated autophagy does not rely on p53 activity. (**a**) Cell extracts from SK-N-BE (2) treated with TCP (1 mM) or SP2509 (1 μM) for 48 h were prepared and probed with specific SESN2 and LC3 antibodies. (**b**) SK-N-BE (2) were treated with different concentrations of SP2509 (0, 0.2, 0.5 and 1 μM) for 48 h and protein extract were prepared and probed with indicated antibody. Actinin was probed as loading control.

It has been recently shown that LSD1 depletion, synergistically with UBE4B inhibition, increases proteasomal and autophagy clearance activating the p53-mediated transcriptional program.^35^ TP53/p53 is involved in the regulation of autophagy through two distinct mechanisms, according to its subcellular localization: cytoplasmic p53 inhibits autophagy through the inhibition of AMPK and the subsequent mTOR activation; nuclear p53 induces the transcription of pro-autophagic genes, including *SESN2*.^36, 37, 38^ To verify that SESN2 upregulation mediated by LSD1 inhibition occurs in a p53-independent manner, we employed SK-N-BE (2) NB cell line, which expresses a non-functional p53.^39^ Cell extracts from SK-N-BE (2) cells treated with TCP, SP2509 or vehicle were processed and assayed for SESN2 expression. As shown in Figure 6a, both inhibitors induce significant increase of SESN2 protein levels, together with a strong induction of LC3-I/LC3-II conversion. Furthermore, we found a SP2509 dose-dependent reduction in phosphorylation of mTORC1 targets, S6p70K and rpS6, confirming that LSD1 inhibition specifically impairs mTORC1 activity (Figure 6b). These findings strongly suggest that p53 function was not essential for SESN2 transcriptional activation and mTORC1 inhibition triggered by pharmacological LSD1 depletion.

### SESN2 is required for autophagy induced by LSD1 inhibition

The findings reported above demonstrated that LSD1 binds and represses *SESN2* gene expression; thus, LSD1 inhibition triggers SESN2 expression. Because SESN2 serves as important regulator of mTORC1 activity, we hypothesized that its upregulation may have a causative role in LSD1-mediated mTORC1 activity modulation and autophagy induction.

**Figure 7:**
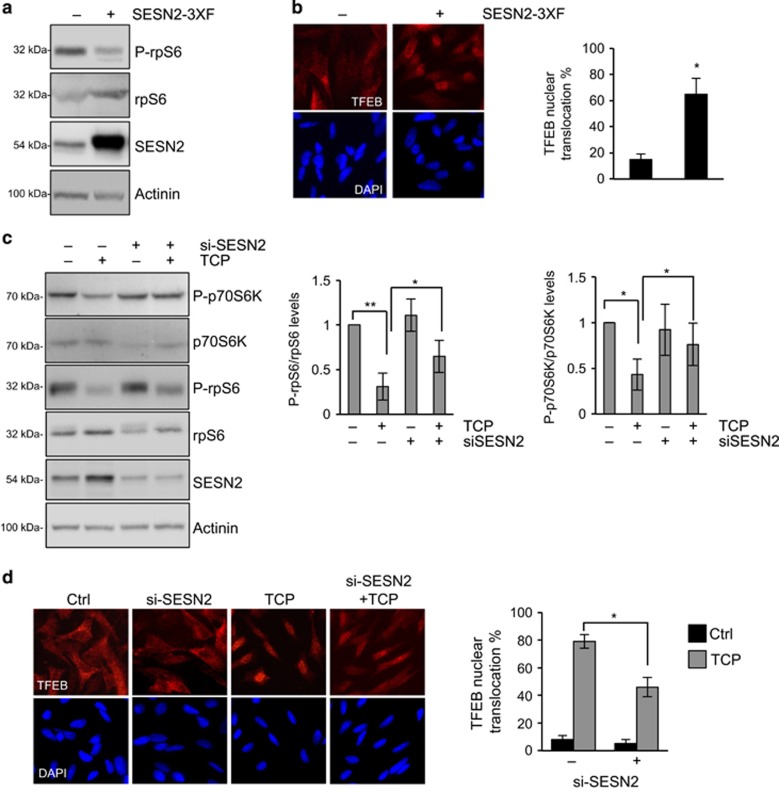
LSD1 regulates autophagy through SESN2 expression. (**a**) SH-SY5Ys were transfected with a SESN2 expression plasmid or empty vector, and protein extracts were prepared and stained with indicated antibody. (**b**) Localization of TFEB was analyzed in cells treated as described in A by immunostaining with TFEB antibody. (**c**) SH-SY5Y cells were transfected with siRNA against SESN2 or scramble. Twenty-four hours after transfection, cells were treated with TCP or vehicle for 24 h. Actinin was probed as loading control. Graphs show quantitative analysis of western blot experiments. A mean value±s.d. of four independent experiments is shown (*P<0.05; **P<0.005). (**d**) SH-SY5Y treated as described in **c** were fixed and stained with anti-TFEB and DAPI. Histograms show the percentage of TFEB nuclear translocation (mean±s.d., *n*=200 cells). *P<0.0001. DAPI, 4',6-diamino-2-phenylindole.

To substantiate the relationship between SESN2 and autophagy induction following LSD1 inhibition, we ectopically overexpressed SESN2 in SH-SY5Y cells, and assessed the effect of increased expression of SESN2 on autophagy. We found that overexpression of SESN2 reduced rpS6 phosphorylation, along with a concomitant increase in TFEB nuclear localization. These findings provide functional evidence that enhanced expression of SESN2 induces autophagy through mTORC1 inhibition and recapitulates the effects of TCP treatment in NB cells (Figures 7a and b). To further define SESN2 role in autophagy induced by LSD1 inhibition, we performed SESN2 knockdown experiments, using a specific siRNA in SH-SY5Y cells, and investigated the functional consequences on LSD1 inhibition in SESN2-silenced cells. Lack of appreciable changes in the phosphorylation level of mTORC1 downstream targets was seen in siRNA-SESN2 cells (Figure 7c). In contrast, reduction of phosphorylation levels of p70S6K and rpS6 induced by TCP treatment is significantly weakened in SESN2-silenced cells; these data suggest that TCP requires SESN2 expression to decrease mTORC1 activity. Moreover, in SESN2 knockdown cells we found a reduced nuclear translocation of TFEB following TCP treatment (Figure 7d), indicating that SESN2 knockdown prevents the LSD1 inhibition-mediated autophagy. Taken together, these results identified SESN2 as a key factor in the autophagy activation mediated by LSD1 inhibition.

### Association of SESN2 expression with clinical outcome in NB patients

**Figure 8:**
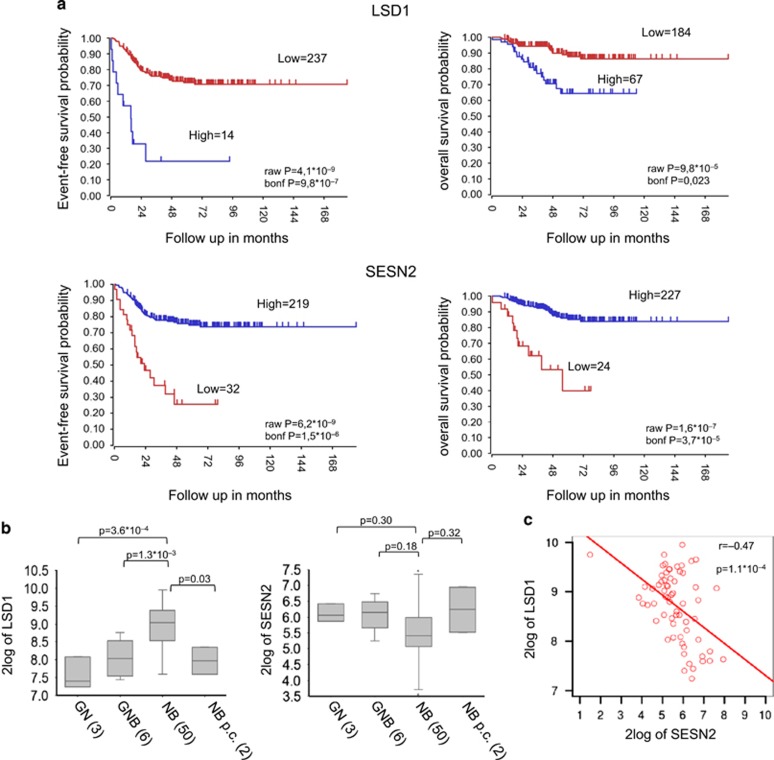
High levels of LSD1 and SESN2 expression are mutually exclusive in NB. (**a**) Kaplan–Meier analysis shows that high *SESN2* gene expression is associated with better survival in NB patients. Patients are grouped by the optimal cutoff of LSD1 and *SESN2* expression. The ‘raw P’ indicates the uncorrected *P*-value, and the ‘bonf P’ indicates the *P*-value corrected for multiple tests according to the Bonferroni method. (**b**) Changes in expression for LSD1 and Sestrin2 in ganglioneuroblastoma (GNB), ganglioneuroma (GN), NB and NB post chemotherapy (NB p.c.). (**c**) Correlation between the expression values of SESN2 and LSD1 in NB tumors (Pearson’s correlation coefficient is shown).

The findings reported above indicated an inverse relationship between the expression of LSD1 and SESN2. It has been shown that high levels of LSD1 expression correlate with undifferentiated NB and adverse outcome.^27^ Our *in vitro* findings imply that in NB patients LSD1 and SESN2 expression levels should be inversely correlated. If so, low expression of SESN2 in NB cancers would be predicted to correlate with poor prognosis. We queried public NB gene expression data repositories (the Oberthuer data set)^40^ for the relative expression levels of LSD1 and SESN2. Accordingly, with previous studies, patients with high levels of LSD1 have poorer prognosis compared with those with low levels. In sharp contrast, high SESN2 expression correlates with better overall and event-free survival (Figure 8a).

Next, we analyzed LSD1 and SESN2 expression in a microarray gene expression data of 61 NBs (GSE12460), of which 50 were NB, 9 were ganglioblastoma and ganglioneuromas, and 2 were NB post chemotherapy. We have recently shown that LSD1 expression is considerably higher in differentiated NBs than in ganglioblastomas and ganglioneuromas.^25^ In contrast, we found that SESN2 expression was lower in NBs (Figure 8b), although the correlation between SESN2 expression in different tumors is only marginally significant. Furthermore, we found an inverse correlation between the expression values of LSD1 and SESN2 in NB tumors (Pearson’s coefficient 0.47; Figure 8c). Collectively, these findings demonstrated that low levels of Sestrins expression correlate with poor prognosis of NB patients, and high levels of LSD1 and SESN2 expression are mutually exclusive in NB.

## Discussion

LSD1 is overexpressed in several types of cancers, and its enhanced expression correlates with more aggressive cancers and poor prognosis. LSD1 is implicated in several biologic processes, such as cell proliferation, epithelial–mesenchymal transition, stem cell pluripotency and differentiation.^24, 25, 26, 27, 41^ However, its involvement in autophagy regulation is still poorly characterized. Here we show that pharmacological and genetic inhibition of LSD1 induces autophagy via the mTORC1-dependent pathway.

The present work highlights a critical role of LSD1 in promoting autophagy in NB cells; we provide for the first time evidence supporting the role of LSD1 as an epigenetic regulator of the autophagic pathway through the modulation of SESN2 expression.

We demonstrated that LSD1 binds and represses the *SESN2* gene; alteration of chromatin structures following LSD1 inhibition leads to the de-repression of the *SESN2* gene, which resulted in a decreased mTORC1 activity. Thus, LSD1 inhibition triggers autophagy, as demonstrated by mTORC1 inhibition, nuclear translocation of TFEB, accumulation of LC3-II and finally the formation of autophagosomes, suggesting a direct link between LSD1-specific transcriptional regulation, mTORC1 cascade and autophagy.

Recent lines of work highlight the involvement of LSD1 in the autophagic machinery. Indeed, in prostate^42^ and ovarian cancer cells^43^ LSD1 inhibition triggers LC3-II accumulation and autophagosome formation; however, the molecular mechanism underlying these effects was not described.

Here, we identify *SESN2* as an LSD1-repressed target gene involved in mTORC1 pathway control. We found that SESN2 promoter is directly bound and repressed by LSD1; pharmacological inhibition of LSD1 triggers a structural modeling in the chromatin surrounding the TSS of the *SESN2* gene, leading to transcriptional activation of SESN2 expression. Moreover, we demonstrated that SESN2-enhanced expression suffices to promote autophagy in NB cells and SESN2 silencing attenuates mTORC1 suppression and autophagy induction by LSD1 inhibition, providing evidence that SESN2 has a critical role in the autophagy activation mediated by LSD1 depletion. SESN2 is a member of the Sestrin family of PA26-related proteins, which has an important role in regulating the cellular response to oxidative stress. TP53/p53 is the master transcriptional regulator of SESN2 under DNA damage and oxidative stress.^38, 44^ Interestingly, it has been recently shown that LSD1 depletion, synergistically with UBE4B inhibition, increases proteasomal and autophagic clearance activating the p53-mediated transcriptional program.^35^ We find that depletion of LSD1 induces activation of SESN2 expression in the SK-N-BE (2) cell line, which express non-functional p53. Thus, LSD1 appears to regulate transcription of SESN2 in both p53-dependent and p53-independent ways.

We recently reported that the MYCN/LSD1 complex inhibits the transcription of the molecular chaperone Clusterin,^41^ which is involved in the autophagic process through the stabilization of the LC3–Atg3 heterodimer, increasing the autophagosome biogenesis and autophagy progression;^45^ we suggest that LSD1 orchestrates a broad-spectrum regulation of the autophagic pathway via transcriptional regulation of several autophagy-related genes.

Autophagy is a catabolic process that, at basal levels, represents the major mechanism for the turnover of cytoplasm components and selective removal of unfolded proteins and damaged organelles. However, autophagy could be activated in response to several stimuli such as oxidative and nutrient stresses, and the mTOR pathway is the main regulator.^12, 36^ Recent studies suggest that the mTORC pathway may be associated with cancer-related epigenetic alterations,^1, 46, 47^ unveiling a key role of the epigenetic network in the autophagy control. HDACI inhibitors have been shown to induce autophagy via FOXO1-dependent pathways;^48^ the methyltransferase EZH2 has been demonstrated to repress several negative regulators of the mTOR pathway and inhibits autophagy.^49^ However, epigenetic role in autophagy regulation and its association with tumorigenesis continue to be uncovered.

The relationship between autophagy and cancer remains controversial. Autophagy seems to have a dual effect in cancer, depending on stage and cell type, and it could act as tumor suppressor or driver of cancer progression.^7, 9^ Some tumors are sensitive to hyperactivation of autophagy; in other circumstances, inhibition of mTORC1 increases cell survival and prevents apoptosis, inducing chemoresistance.^50^ Although relationship between autophagy and tumor progression is disputed, during early stages of carcinogenesis autophagy seems to suppress tumor initiation and cancer development is often associated with defective autophagy.^7, 9^ We cannot exclude that LSD1 overexpression may contribute to tumor initiation by suppressing the expression of key regulators of autophagy induction, although further studies are required to clarify this issue. Interestingly, analysis of publicly available expression data of large number of NBs highlighted that high SESN2 expression correlates with better overall and event-free survival. Interestingly, high levels of LSD1 and SESN2 expression are mutually exclusive in NB tumors. Collectively, these findings support and corroborate the broad significance of our *in vitro* results.

In conclusion, data reported here establish the critical role of LSD1 in autophagy and indicate that, in NB cells, LSD1 knockdown induces autophagy through the SESN2–mTORC1 pathway. Our results strongly support the concept that LSD1-dependent epigenetic alterations may influence the expression of autophagy-related genes and provide a novel link among epigenetic regulation, mTOR pathway and tumorigenesis.

## Materials and methods

### Cell culture and treatments

SH-SY5Y and SHEP Tet-21/N cells were cultured in Dulbecco's Modified Eagle Medium supplemented in 10% fetal bovine serum. SK-N-BE (2) was cultured in 1:1 mixture dulbecco's modified eagle medium/F-12 containing 10% fetal bovine serum. When indicated, cells were treated with TCP (1 mM, Enzo Life Sciences, Farmingdale, NY, USA) or SP2509 (0.2/0.5/1 μM, Cayman Chemical Company, Ann Arbor, MI, USA) for 6, 12, 24 or 48 h. An amount of 20 mM of NH_4_Cl was administrated for 24 h to block autophagic flux.

### Transfection and silencing

For transient transfections of Tet-21/N and SH-SY5Y, cells cultured on 100-mm dishes were transfected with 12 μg of SESN2 plasmid, GFP-mRFP-LC3 (kind gift from Dr A Fraldi, Tigem Institute) construct or empty vector using Lipofectamine 2000. For LSD1 knockdown, 50 or 100 nM siRNA targeting LSD1 or LSD2 (GE Dharmacon, Lafayette, CO, USA) or scramble were transfected in Tet-21/N cells using a MicroPorator Digital Bio Technology(Waltham, MA, USA), according to the recently described protocol.^41^ Briefly, 2x10^6^ cells were collected by trypsin/EDTA digestion, washed once with calcium and magnesium-free phosphate-buffered saline (PBS) and resuspended in 100 μl of resuspension buffer, mixed with siRNA or scramble and electroporated as described.^25^ The efficiency of LSD1 siRNA knockdown was assayed 48 h after transfection by western blotting. For silencing assays, 45 nM siRNA targeting SESN2 (GE Dharmacon) or scramble were transfected in SH-SY5Y cells using Viromer Green; Cells were collected for analysis 48 h after transfection.

### PathScan assay

For PathScan assay (PathScan Intracellular Signaling Array Kit, Cell Signaling, Danvers, MA, USA), cell lysates from Tet-21/N cells, treated with TCP or vehicle for 24 h, were prepared according to the manufacturer's protocol; 75 μl of lysates was added to each well previously prepared with Blocking Buffer and the slide was incubated for 2 h at room temperature. After incubation, the slide was washed four times with Wash buffer and each well was incubated with 75 μl of Detection Antibody Cocktail for 1 h at room temperature. Then, the slide was washed three times and incubated for 30 min with 75 μl of horseradish peroxidase-linked Streptavidin reagent. Next, the slide was washed, incubated with Lumi Glo/peroxide solution and displayed by biochemiluminescence acquisition. Protein expression levels were quantified and normalized by positive controls using ImageJ32 software (National Institutes of Health, Bethesda, MD, USA).

### Protein extraction and western blot

Whole-cell extracts were prepared with buffer F (10 mM Tris HCl pH 7.5, 150 mM NaCl, 30 mM Na_4_O_7_P_2_, 50 mM NaF, 5 mM ZnCl_2_, 0.1 mM Na_3_VO_4_, 1% Triton, 0.1 mM PMSF). Fifty micrograms of protein extracts were separated by SDS–PAGE and indicated antibodies are listed in Supplementary Table S1. Full scans of western blot data are in Supplementary Figure 3.

### Immunofluorescence and microscopy

Immunofluorescence assay was performed as previously described.^25^ Overall, 2 × 10^4^ cells were plated in a 12-well plate, in which a coverslip had been placed and treated as indicated. Cells were then washed, fixed in 4% paraformaldehyde in PBS, permeabilized in 0.1% Triton X-100 in PBS for 5 min, pre-blocked in 2% bovine serum albumin for 30 min and incubated at 37 °C for 1 h with rabbit anti-TFEB. Cells were then incubated for 30 min at room temperature with Cy3-coniugated secondary antibody and nuclei were stained with 4',6-diamino-2-phenylindole (DAPI). Nikon Eclipse TE 2000-U microscope (Nikon, Shinjuku, Japan) was used for image acquisition. Quantitative TFEB nuclear translocation analyses were performed by ImageJ32 software, calculating the ratio value resulting from the average of nuclear TFEB signal intensity divided by the average of the cytosolic TFEB signal intensity, normalized on negative and positive control samples. In case of GFP-mRFP-LC3 experiments, cells were just fixed in 4% paraformaldehyde and images were collected using a laser-scanning microscope (LSM 510 META, Carl Zeiss Microimaging Inc., Oberkochen, Germany) equipped with a Plan Apo × 63 oil immersion (numerical aperture 1.4) objective lens. Moreover, we acquired the images with the same setting (laser power and detector gain) as well as we kept the same threshold of fluorescence intensity in all experimental conditions. Quantification analyses were carried out using LSM 510 software. We evaluated the autophagic flux counting the number of green and red puncta per cell (number of cells >100). Student’s *t*-test was used to determine the significance and error bars show the s.d. of the average.

### RNA extraction and qRT–PCR

Total RNA was extracted from Tet-21/N cells and reverse transcription reaction was performed using Quantitec Reverse Transcription Kit (Qiagen, Hilden, Germany). cDNA thus obtained was analyzed by qPCR using SYBR Green 2X PCR Master Mix (Applied Biosystem, Waltham, MA, USA). Each sample was run in triplicate and the expression of housekeeping beta-glucoronidase (GUSb) gene used for normalization as described.^41^ Primers are presented in Supplementary Table S2.

### Chromatin immunoprecipitation assay

ChIP assays were performed as described.^25^ In all, 1 × 10^7^ cells treated as indicated were crosslinked using 1% formaldehyde. Cell pellet was lysed and sonicated into 200-bp fragments by using Bioruptor (Diagenode, Liege, Belgium). An aliquot of sonicated material was used as input. Remaining samples were incubated overnight with antibodies listed in Supplementary Table S1; immunoprecipitated DNA was purified and quantified by qPCR with the primer sets described in Supplementary Table S2 and normalized to input DNA.

### Survival analysis and association with NB stages by gene expression studies

Kaplan–Meier curves were calculated for Oberthuer public gene expression data^40^ using the R2 web tool (http://r2.amc.nl) as described.^51^ Briefly, the optimal cutoff for each gene was determined by R2 package to generate the Kaplan–Meier curves, and significance (raw P) and the *P*-value (Bonferroni-corrected) were calculated. Another set of gene expression data of 61 tumors (GEO ID: GSE12460) including 50 NB, 6 ganglioneuroblastoma, 3 ganglioneuroma and 2 NB post chemotherapy was downloaded, and the Mann–Whitney test was used to test statistical significance of differences in gene expression among groups. The correlation between gene expression between Sestrins and LSD1 was evaluated by Pearson correlation in 64 NBs.

### Statistical analysis

All experiments were repeated at least two times. Graphs representing data express mean±s.d. Statistical significance was calculated by unpaired, two-tailed Student's *t*-test. *P*<0.05 was considered statistically significant.
