# Supplementary material for: Lysine-specific demethylase LSD1 regulates autophagy in neuroblastoma through SESN2-dependent pathway
Source: Oncogene. 2017 Aug 7;36(48):6701–11. doi: 10.1038/onc.2017.267 (PMC5717079; doi:10.1038/onc.2017.267)
Supplement: Supplementary Figure 1 [file onc2017267x1.pdf]

Supplementary Figure 1

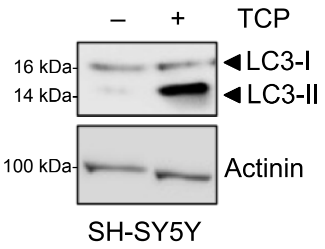

Supplementary Figure 1: SH-SY5Y cells were treated with TCP for 24 h and protein extract were prepared and probed using anti-LC3 antibody. Actinin was probed as loading control.
