# Supplementary material for: Lysine-specific demethylase LSD1 regulates autophagy in neuroblastoma through SESN2-dependent pathway
Source: Oncogene. 2017 Aug 7;36(48):6701–11. doi: 10.1038/onc.2017.267 (PMC5717079; doi:10.1038/onc.2017.267)
Supplement: Supplementary Figure 2 [file onc2017267x2.pdf]

Supplementary Figure 2

A

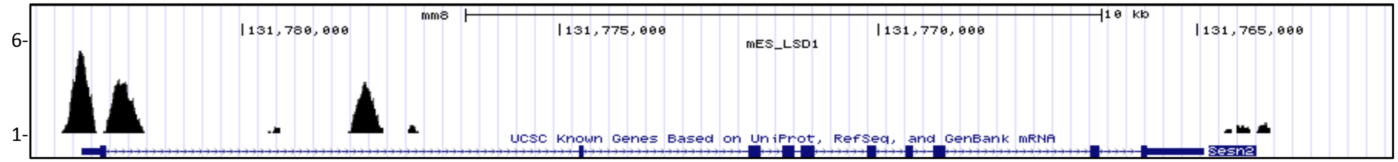

B

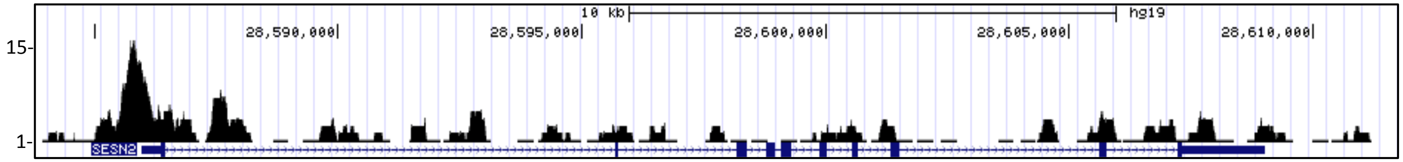

Supplementary Figure 2: Screenshots of UCSC genome browser of SESN2 gene are shown. (A) and (B) the peaks represent the LSD1 binding on the promoter region and TSS of SENS2 gene in mouse and human cells respectively.
