# Supplementary material for: Lysine-specific demethylase LSD1 regulates autophagy in neuroblastoma through SESN2-dependent pathway
Source: Oncogene. 2017 Aug 7;36(48):6701–11. doi: 10.1038/onc.2017.267 (PMC5717079; doi:10.1038/onc.2017.267)
Supplement: Supplementary Figure 3 [file onc2017267x3.pdf]

Supplementary Figure 3

Fig. 1b (*)

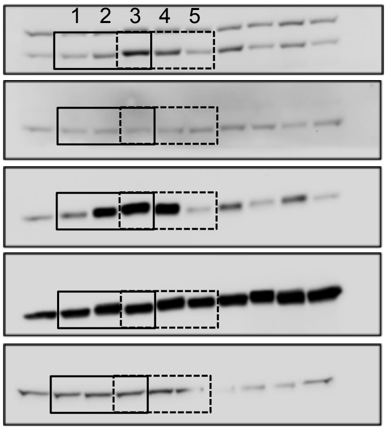

Fig. 1c

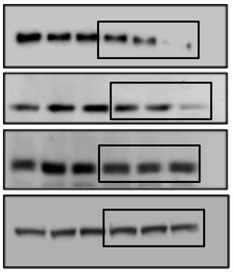

Fig. 1d

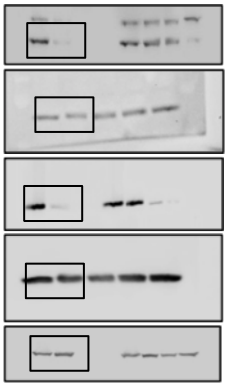

Fig. 3a

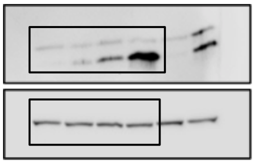

Fig. 3b

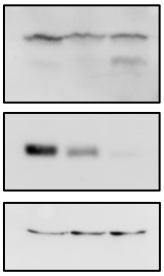

Fig. 4a

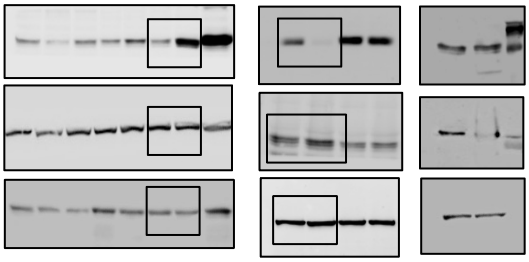

Fig. 5a

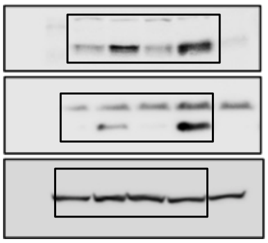

Fig. 5b

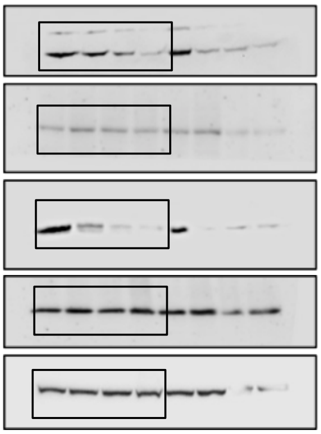

Fig. 6a

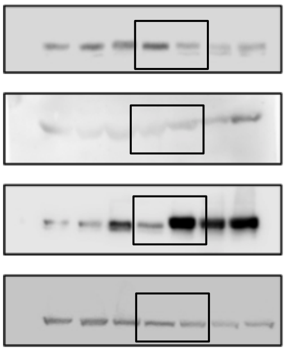

Fig. 6c

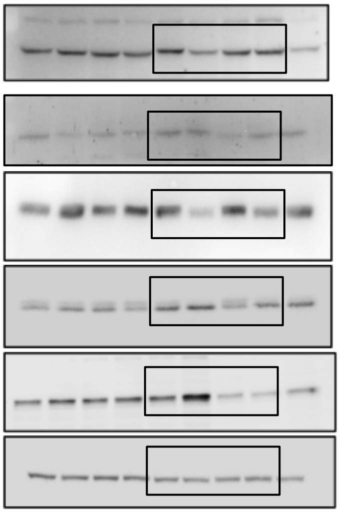

Supplementary Figure 3: Full scans of Western data. (*) Only in Fig. 1B, for better results presentation, lanes have been separated into two panels that share the control sample (lane 3).
