# Supplementary material for: Lysine-specific demethylase LSD1 regulates autophagy in neuroblastoma through SESN2-dependent pathway
Source: Oncogene. 2017 Aug 7;36(48):6701–11. doi: 10.1038/onc.2017.267 (PMC5717079; doi:10.1038/onc.2017.267)
Supplement: Supplementary Table 1 [file onc2017267x5.pdf]

Table S1: antibodies used in this work

Protein	Used for	Manufacturer
Actinin	WB	sc-17829, Santa Cruz
Sestrin 2	WB	10795-1-AP, Proteintech
Phospho-S6 Ribosomial Protein	WB	2215, Cell Signaling
S6 Ribosomial Protein	WB	2217, Cell Signaling
Phospho-p70 S6 Kinase (Thr 389)	WB	9205, Cell Signaling
p70 S6 Kinase	WB	2708, Cell Signaling
LC3A/B	WB	4108, Cell Signaling
LSD1	ChIP, WB	ab17721, Abcam
TFEB	IF	4240, Cell Signaling
H3Ac	ChIP	06-599, Millipore
H3K27me3	ChIP	07-449, Millipore
H3K4me2	ChIP	ab32356, Abcam
H3K9me2	ChIP	ab1220, Abcam
LSD2	WB	Ab 193080, Abcam
