# Supplementary material for: Lysine-specific demethylase LSD1 regulates autophagy in neuroblastoma through SESN2-dependent pathway
Source: Oncogene. 2017 Aug 7;36(48):6701–11. doi: 10.1038/onc.2017.267 (PMC5717079; doi:10.1038/onc.2017.267)
Supplement: Supplementary Table 2 [file onc2017267x6.pdf]

Table S2: oligos used in this work

	Gene	FW	Rev
qChiP	SESN2 (–10Kb)	CCAAGTTGTGAATGCAAAGG	AGCCGAGATCAGGCCACT
qChiP	SESN2 (TSS)	AGTCCCTCCAGGAACTGAAA	GTCATTAGGGTTGCGTGATG
qRT-PCR	SESN2	GTGGACACCTCCGTGCTC	GGTTCACCTCCCCATAATCA
qRT-PCR	GUSb	GTGGGCATTGTGCTACCTC	ATTTTTGTCCCGGCGAAC
qRT-PCR	SESN1	GGGCCGTTACCCCTACATTA	TTCACTAAGTAGGAGCACTGATGTC
qRT-PCR	SESN3	GCTAATGACAACAACATAGAGAATGC	CTCTAGCTCACTTAGAGAATCCACAA
qRT-PCR	LSD2	CCACAATAAATCAGTCATCATTATCG	TCTTTGGCTTCCAGGACAGT
qRT-PCR	LSD1	AGACGACAGTTCTGGAGGGTA	TCTTGAGAAGTCATCCGGTCA
